# Multidetector computed tomography utilization in an urban sub-Saharan Africa setting: user characteristics, indications and appropriateness

**DOI:** 10.11604/pamj.2020.37.42.21176

**Published:** 2020-09-09

**Authors:** Joshua Tambe, Lawrence Mbuagbaw, Georges Nguefack-Tsague, Joseph Foyet, Pierre Ongolo-Zogo

**Affiliations:** 1 Post-Graduate School for Life Sciences, Health and Environment, The University of Yaoundé I, Yaoundé, Cameroon,; 2 Centre for the Development of Best Practices in Health (CDBPH), Yaoundé Central Hospital, Yaoundé, Cameroon,; 3 Division of Radiology, University of Buea, Buea, Cameroon,; 4 Department of Health Research Methods, Evidence and Impact, McMaster University, Hamilton, Canada,; 5 Biostatistics Unit, Father Sean O'Sullivan Research Centre, St. Joseph's Healthcare, Hamilton, Canada,; 6 Biostatistics Unit, Department of Public Health, Faculty of Medicine and Biomedical Sciences, The University of Yaoundé I, Yaoundé, Cameroon,; 7 Deido Radiology and Ultrasonography Centre, Douala, Cameroon

**Keywords:** Multidetector computed tomography, utilization, appropriateness

## Abstract

**Introduction:**

multidetector computed tomography (MDCT) is a widely used cross-sectional imaging modality despite increasing concerns about radiation exposure and overuse. The aim of this study was to describe the socio-demographic characteristics of MDCT users in an urban city in Cameroon and to assess the clinical indications for appropriateness.

**Methods:**

we conducted a survey of MDCT users and collected data on demographic attributes and socialization patterns, clinical indications for MDCT and time to obtain MDCT. MDCT appropriateness was assessed using the American College of Radiologists Appropriateness Criteria®. Frequencies, percentages, odds ratios and 95% confidence intervals were used to summarize the data.

**Results:**

with a response rate of 79%, 511 MDCT users were surveyed. The mean (standard deviation) age was 45(19) years and male to female sex ratio 1:1. Seventy-eight percent (95% confidence interval [CI]: 74-83%) of respondents reported not having any health insurance. Head scans accounted for 52% (95%CI: 47-56%) of all scans with trauma (19% [95%CI: 15-22%]), low back pain (18% [95%CI: 14-21%]) and suspected stroke (10% [95%CI: 7-13%]) being the most frequent indications. Sixteen percent (95%CI: 13-20%) of the scans were judged to be inappropriate. Predictors of MDCT appropriateness after multivariable logistic regression modeling were age (aOR=0.97; P=0.009; 95%CI=0.94-0.99), health insurance ownership (aOR=0.40; P=0.034; 95%CI=0.18-0.94) and being referred by non-specialist physicians (aOR=0.20; P<0.001; 95%CI=0.09-0.47).

**Conclusion:**

people from all social strata use MDCT, mostly appropriately and especially for head scans after trauma in this urban setting. However, the proportion of inappropriate studies was considerable suggesting the need for control measures.

## Introduction

Advances in medical technology have had a significant impact in improving the health outcomes for many patients [1]. Medical imaging has benefitted much from such technological developments with improved detection and surveillance of disease or injury and also providing less invasive alternative methods to surgery in the treatment of some conditions [1]. Computed tomography (CT) for example is an imaging modality that has known a lot of improvements in the past two decades. The excellent spatial and temporal resolution of multidetector computed tomography (MDCT), improved anatomic detail, sophisticated image post-processing algorithms and availability for use in emergency situations have widened the scope and increased the frequency of its use [2,3]. Unfortunately sophisticated MDCT technology has come at a price: the cost of health care has soared and overutilization has increased [1,4]. In developing countries like Cameroon where an estimated 40% of the population live below the poverty line and up to 96-98% do not have any form of financial protection for health [5], it would be fair to say that using MDCT would be a challenge with out-of-pocket (OOP) payments constituting the main gateway to accessing MDCT. This would definitely constitute a potential barrier to fully benefitting from available health care services whereas access to health care is a cornerstone in the walk towards achieving universal health coverage [6].

MDCT being an imaging modality that uses x-rays, a form of ionizing radiation, there are radiation exposure risks. X-rays absorbed by the body can produce free radicals that can alter the structure of genes and result in mutations or radiation-induced cancer. MDCT scanning has been found to deliver far more radiation exposure to x-rays compared to conventional radiography and there have been calls to caution its use [7-9]. It is worthy of note that the rational use of MDCT will limit overutilization and the wasteful use of resources which are first and foremost scarce. Healthcare services are geographically inequitably distributed in Cameroon with the concentration of specialists and sophisticated equipment in tertiary healthcare facilities in the major cities [5]. In an attempt to improve access to healthcare services the government has been creating medical imaging centers with MDCT technology throughout the country. This action may however have unintended consequences such as inappropriate use especially if some regulatory measures are not considered. Also MDCT use by those in need might still not be guaranteed despite its availability as other factors such as affordability have a role to play. Understanding therefore the social and demographic characteristics of MDCT users, the clinical indications and their appropriateness can be useful in informing policies to improve access.

## Methods

**Study setting:** the study was carried out at Deido Radiology and Ultrasonography Centre, a medical imaging facility situated at the heart of the economic capital of Cameroon, Douala. This facility is privately owned and well-known locally with close to eighteen years of existence. It receives clients from both public and private health facilities for imaging studies and the cost of the studies is comparable to that of the public referral hospitals. A wide range of imaging technologies are available at this center including ultrasonography, Doppler studies, conventional radiography and contrast studies, mammography, dental pantomography and cone beam technique, MDCT and magnetic resonance imaging (MRI). Besides physical accessibility and the broad range of imaging services offered, another major attraction to this center is the fact that often the results are obtained on the same day the imaging study is performed, a situation that is convenient for many clients. At the time we collected data there were three radiologists working in shifts, five radiology technologists and three medical secretaries amongst others.

**Participants and data collection:** we carried out face-to-face structured interviews of consecutive and consenting MDCT users or caregivers for persons who could not provide the information needed. Consent to participate in the study was either verbal or written and institutional ethical approval was obtained (reference number 001-08/2017/CEI_CERAD). Cochran´s method for surveys was used to estimate a minimum sample size [10]. Assuming an alpha level of 0.05 and a 5% margin of error, a population variance estimate of 0.25 with MDCT appropriateness as primary outcome (measured as a binary variable), we hypothesized a non-response rate of 20% and the estimated minimum sample size was 373 participants. Data were collected for a period of ten months from October 2017 to July 2018. Information was collected on demographic attributes (age and gender), social and professional characteristics (educational achievement, marital status, occupation, socioeconomic status, ownership of a financial protection for health scheme) and clinical indications. Data were also collected on the anatomic region scanned, the date the scan was requested and date performed and on the qualification of the prescribing healthcare provider. Socioeconomic status (SES) was assessed using household amenities score, a tool that indicates the living conditions of the respondents and which had been used in a previous study in a similar setting [11]. The scores derived from all of the respondents were then divided into quintiles and each respondent assigned to the quintile corresponding to their score. MDCT appropriateness was assessed using the American College of Radiologists (ACR) Appropriateness Criteria® available online [12].

These appropriateness criteria are guidelines based on evidence put together by experts from different clinical specialties. The main goal for these guidelines is to assist referring healthcare providers request for the most appropriate imaging study for specific clinical situations. For example the clinical condition “moderate or severe acute closed head injury (Glasgow coma scale <13)” has as recommendation “CT scan of the head without intravenous contrast” and the strength of the recommendation is 9, on a scale from 1 to 9. This would mean head CT without contrast is the most appropriate study for this clinical condition. Another clinical situation such as “low back pain with suspected cauda equina syndrome or rapidly progressive neurologic deficit” has “MRI of the lumbar spine without intravenous contrast” as the most appropriate study with a score of 9. A trained research assistant fluent in French, English and the local pidgin-English language collected information from all the participants during the study period. Request forms for MDCT were used to retrieve information participants could not provide. Before the start of data collection, a meeting was held with the staff of the study site to devise a means of data collection that would not interfere with the flow of activities at the facility. An identification badge was handed to the research assistant alongside a uniform similar to that worn by the staff of the facility. The principal investigator (JT) cross-checked all the data forms after data collection and assessed the clinical indications for appropriateness. Appropriateness for the study was consensually agreed by JT and POZ.

**Data analysis:** the information on the data forms were transcribed onto a Microsoft Excel® spreadsheet and analyzed using Stata®12 (StataCorp, Texas, USA). Frequencies, percentages and 95% confidence intervals (CI) were used to summarize categorical data whilst continuous data were summarized using the mean (standard deviation) and median (range). The data is presented using tables. Univariate and multivariable linear regression techniques were used to verify if any factor was associated with the time to obtain MDCT. Binary logistic regression with multivariable modeling were also used to verify if any characteristic was associated with MDCT appropriateness. In the multivariable regression models, the covariates were selected based on the literature and the authors´ expectations. These covariates were entered as a block and included age, gender, level of education, socioeconomic status, health insurance ownership and quality of the referring healthcare provider. All P-values were two-tailed with a 0.05 significance threshold. Model fit was assessed using the R^2^ statistic.

## Results

We surveyed 511 MDCT users representing 79% of all persons we approached for potential participation in the study.

**Demographic attributes and socialization patterns:** the mean (standard deviation; SD) age of the respondents was 44.5 (19.1) years with range from 1 to 87 years. The male to female sex ratio was 1:1. Table 1 summarizes the educational achievement of the respondents, socioeconomic status, health insurance ownership and occupation of the respondents, whilst Table 2 presents the quality of referring healthcare providers and the body region scanned.

**Table 1:** social characteristics of respondents

Characteristic	Frequency (%; 95% CI)
**Educational achievement (N=426)**	
None completed	29 (6.8; 4.7-10.1)
Completed primary	118 (27.7; 25.3-34.6)
Completed secondary	193 (45.3; 43.9-54.0)
Completed university	86 (20.2; 17.6-26.0)
**Socioeconomic status (N=468)**	
SES quintile 1	93 (19.9; 19.3--27.9)
SES quintile 2	96 (20.5; 20.0-28.7)
SES quintile 3	92 (19.7; 19.0-27.7)
SES quintile 4	112 (23.9; 23.8-33.0)
SES quintile 5	75 (16.0; 15.0-23.0)
**Health insurance ownership (N=394)**	
Yes	85 (21.6; 17.4-25.8)
No	309 (78.4; 74.2-82.6)
**Employment status (N=381)**	
Worker (employed with formal contract, employed informally, self-employed)	286 (75.1; 70.7-79.4)
Retired	27 (7.1; 4.5-9.7)
Unemployed / housewife	25 (6.6; 4.1-9.0)
Pupils / students	43 (11.3; 8.1-14.5)

**Table 2:** qualities of referring healthcare providers and anatomic region scanned

Characteristic	Frequency (%; 95% CI)
**Referring healthcare provider (N=406)**	
Specialist doctor	204 (50.1; 45.2-55.2)
Physician, general practitioner	193 (47.4; 42.6-52.6)
Nurse	5 (1.2; 0.0-2.4)
Medical student	2 (0.5; 0.3-1.3)
Self-referral	2 (0.5; 0.3-1.3)
**Body region scanned (N=502)**	
Abdomen & pelvis	55 (10.9; 8.1-13.8)
Chest	18 (3.6; 1.9-5.3)
Head & facial bones	259 (51.6; 47.1-56.1)
Lumbar spine	99 (19.7; 16.1-23.3)
Paranasal sinuses	18 (3.6; 1.9-5.3)
Others^a^	53 (10.6; 7.8-13.3)

a Others include multiple body regions, pelvimetry, cervical and dorsal spine, temporal bone, joints and extremities, nasopharynx and myelography; CI, confidence interval

**Indications for multidetector computed tomography scans:** indications or clinical information were written on the request forms of 432 participants out of 511 (84.5%). Trauma was the most frequent reason for requesting a scan (19%; 95%CI=15-22%). Table 3 summarizes indications for MDCT scan per anatomic region.

**Table 3:** indications for MDCT scan by anatomic region

Indications for MDCT scan^a^	Frequency (%; 95% CI)
**Head & facial bones**	
Trauma	95 (19.0; 15.4-22.5)
Suspected stroke	51 (10.2; 7.4-12.9)
Headaches (persistent, chronic)	39 (7.8; 5.3-10.2)
Others (suspected intracranial space-occupying lesion, convulsion/epileptic seizure, etc.)	74 (14.7; 11.6-17.8)
**Lumbar spine**	
Pain (lower back pain), sciatica, lumbago, intermittent claudication, suspected hernia	88 (17.5; 14.1-21)
Trauma	3 (0.6; 0.2-1.4)
Others (Pott's disease, osteo-arthritis, etc.)	8 (1.6; 0.5-2.7)
**Abdomen & pelvis**	
Mass, suspected tumor	18 (3.6; 1.9-5.3)
Urinary tract problems (hydronephrosis, calculi, polyuria, obstruction, tumor)	15 (3.0; 1.4-4.6)
Abdominal pain	7 (1.4; 0.4-2.4)
**Chest**	
Suspected tumor/mass	7 (1.4; 0.4-2.4)
Chronic cough	2 (0.4; 0.2-0.9)
Others (include trauma, repeat scans, chest pain, e.t.c.)	9 (1.8; 0.6-3.0)

a Analysis done for 416 respondents; MDCT, multidetector computed tomography.

**Time to multidetector computed tomography scan utilization:** the median time to MDCT scan utilization was 3 days (range: 0 to 18). The second quintile of socioeconomic status was associated with a longer time to MDCT scan utilization compared to the first SES quintile in both the univariate and multivariable linear regression modeling (beta coefficient=0.19, 95%CI=-11.07-11.46; P=0.019). Referral by a specialist physician was associated with a longer time to MDCT utilization in the univariate analysis only. The linear regression analysis is presented in Table 4.

**Table 4:** univariate and multivariate analysis of time to MDCT utilization

Variables	Univariate		Multivariate	
	Beta coefficient^a^ (95% CI)	P-value	Beta coefficient^a^ (95% CI)	P-value
Age (years, N=496)	-0.08 (-0.21, 0.05)	0.079	0.01 (-0.19, 0.22)	0.844
**Gender (N=488)**				
Female	ref		ref	
Male	0.04 (-4.87, 4.96)	0.334	0.13 (-6.65, 6.9)	0.051
**Education (N=425)**				
None	ref		ref	
Completed primary	-0.09 (-9.95, 9.78)	0.352	-0.16 (-13.83, 13.50)	0.160
Completed secondary	0.01 (-9.48, 9.49)	0.960	-0.09 (-13.49, 13.30)	0.449
Completed university	-0.06 (-10.28, 10.16)	0.477	-0.14 (-15.39, 15.12)	0.228
**Socioeconomic status (N=465)**				
Quintile 1	ref		ref	
Quintile 2	0.16 (-7.91, 8.23)	0.006	0.19 (-11.07, 11.46)	0.019
Quintile 3	0.04 (-8.12, 8.19)	0.529	0.07 (-11.26, 11.40)	0.425
Quintile 4	0.01 (-8.10, 8.17)	0.812	0.04 (-11.26, 11.34)	0.654
Quintile 5	0.03 (-8.10, 8.16)	0.625	0.02 (-11.48, 11.52)	0.841
**Health insurance (N=339)**				
Yes	0.02 (-6.93, 6.98)	0.656	0.09 (-8.35, 8.52)	0.178
No	ref		ref	
**Healthcare provider (N=479)**				
General physician	ref		ref	
Specialist doctor	0.13 (-4.98, 5.23)	0.006	0.12 (-6.87, 7.12)	0.065
Nurse	-0.01 (-21.07, 21.04)	0.758	0.00 (-31.46, 31.46)	0.967
Medical student	-0.01 (-27.68, 27.65)	0.755	0.00 (-31.79, 31.79)	0.988
Self-referral	0.00 (-27.66, 27.66)	0.975	0.01 (-53.58, 53.60)	0.884
**MDCT appropriateness^b^ (N=432)**				
Yes	-0.01 (-7.39, 7.38)	0.903	0.06 (-8.98, 9.09)	0.377
No	ref		ref	

a Beta coefficient from linear regression model; ^b^ The American College of Radiologists uses three categories which include “appropriate”, “may be appropriate” and “not appropriate”. The first two categories were merged as “Yes” for the above and subsequent analysis; CI, confidence interval; ref, reference category; Model-adjusted R2 = 0.010; P-value=0.291

**Appropriateness of multidetector computed tomography scan utilization:** of the 432 scans that could be categorized for appropriateness, 71 were judged to be inappropriate (16.4%; 95%CI=12.8-20.1). Fifteen percent (73 out of 505) of the scans could not be classified for appropriateness due to lack of sufficient clinical information. Predictors of MDCT appropriateness after multivariable logistic regression modeling were age (aOR=0.97; P=0.009; 95%CI=0.94-0.99), health insurance ownership (aOR=0.40; P=0.034; 95%CI=0.18-0.94) and a non-specialist physician referral (aOR=0.20; P<0.001; 95%CI=0.09-0.47). The findings of the logistic regression analysis are presented in Table 5.

**Table 5:** multidetector computed tomography scan appropriateness

Variables	Univariate		Multivariate	
	Crude OR (95% CI)	P-value	Adjusted OR (95% CI)	P-value
Age (years, N=421)	0.98 (0.97, 0.99)	0.038	0.97 (0.94, 0.99)	0.009
**Gender (N=416)**				
Female	1		1	
Male	1.61 (0.95, 2.70)	0.074	1.41 (0.68, 2.92)	0.350
**Education (N=368)**				
None	1		1	
Completed primary	2.64 (0.97, 7.14)	0.056	3.90 (1.06, 14.33)	0.040
Completed secondary	2.27 (0.89, 5.75)	0.084	3.23 (0.96, 10.84)	0.057
Completed university	1.58 (0.58, 4.31)	0.368	1.91 (0.45, 8.16)	0.383
**Socioeconomic status (N=400)**				
Quintile 1	1		1	
Quintile 2	0.60 (0.25, 1.41)	0.241	0.24 (0.06, 0.89)	0.034
Quintile 3	0.65 (0.27, 1.59)	0.345	0.32 (0.08, 1.27)	0.105
Quintile 4	0.72 (0.29, 1.74)	0.460	0.46 (0.12, 1.74)	0.255
Quintile 5	0.59 (0.25, 1.39)	0.228	0.54 (0.14, 2.09)	0.373
**Health insurance (N=339)**				
Yes	0.56 (0.29, 1.08)	0.085	0.40 (0.18, 0.94)	0.034
No	1		1	
**Healthcare provider (N=414)**				
Specialist doctors	1		1	
Others	0.35 (0.19, 0.62)	<0.001	0.20 (0.09, 0.47)	<0.001

Model P-value <0.001; OR, odds ratio; CI, confidence interval

## Discussion

MDCT use in the study context spans across all age groups and there isn´t any gender predilection. The proportion of MDCT users without health insurance ownership was lower than the reported 96-98% in a national survey [5]. This may be explained by the fact that the study was carried out in a private health facility in an urban setting and therefore does not reflect the reality in the national territory. Also people with some form of financial protection for health are more likely to self-refer or put pressure on their doctors to prescribe a diagnostic imaging modality [13], hence a higher percentage of MDCT users with insurance ownership in this study. MDCT imaging of the head and facial bones accounted for slightly more than half of all the scans. MDCT scan is the first-line imaging modality recommended for head trauma and this clinical indication was the most frequent. It is worth noting that commercial motor-bikes are a major means of transportation in the city of Douala and many ply this trade without any license to certify some basic training. There is often more than one passenger and no helmet for the rider and his passengers, increasing the risk and severity of head injury when an accident occurs and this would often require an imaging investigation. To further explain the increased request for head MDCT scans, MDCT is being utilized in many clinical situations where MRI is most appropriate especially in otolaryngology and neuroimaging due to the fact that MRI is still relatively expensive.

Socioeconomic status (SES) is reportedly associated with the use of health care services with more specialist consultations and medical imaging use amongst people with a high SES [14,15]. In this study, a low SES was associated with the time to obtain MDCT. However, the proportion of MDCT users did not differ according to SES as reported in other studies [16,17]. Given that trauma mostly due to road traffic accidents, suspected stroke and varying degrees of pain constitute medical emergencies, it is likely patients and family members are more committed to overcome the barrier of out-of-pocket payments and get MDCT done when requested. A study carried out in a setting where financial protection for health services is available for the majority of the population reported that SES did not influence MDCT use when the clinical indications were without ambiguity [18].

Specialist doctors prescribed most of the MDCT scans compared to other categories of referring healthcare providers and more appropriately too. The high concentration of specialist physicians in urban settings could explain the significant proportion of MDCT requests. Also appropriate MDCT requests by specialist physicians can be attributed to better mastery of the indications for MDCT as a result of further medical training.

The relatively high cost of MDCT in this private health facility (109 to 218 USD) compared to government-owned facilities, the burden of OOP payments for services and ionizing radiation exposure from MDCT are all factors pointing towards a rational use of MDCT. The proportion of scans judged to be inappropriate using the American College of Radiologists (ACR) Appropriateness Criteria® was considerable, indicating unnecessary radiation exposure and probably some wasteful use of resources. In a study carried out in South Africa [19], inappropriate computed tomography and MRI scans use accounted for 6.4% using the ACR Appropriateness Criteria®. However, this study did not report the proportion of inappropriate scans specific to computed tomography.

To explain the inappropriate MDCT requests, studies have shown that referring clinicians are not versed with MDCT indications neither are many aware of the ionizing radiation clients are exposed to [20,21]. A high workload may also be responsible as getting the scan done saves time for a detailed clinical examination. In Cameroon, there is no system of prior approval of MDCT requests by any competent body or individual before they are performed and there are no restrictions as to who can request for MDCT. This situation is likely to increase the number of inappropriate studies requested.

In this study, an association is reported between health insurance ownership and an increased likelihood of inappropriate MDCT utilization. It is expected that health insurance ownership will abolish or minimize out-of-pocket payments for health care services thereby improving access. However, over-utilization of medical imaging appears to be an unintended consequence of health insurance ownership. Clients with health insurance might be predisposed to pressure healthcare providers to request for medical imaging studies even when these might not be necessary. Also given that the study was carried out in a private facility, it is possible obtaining MDCT might be facilitated for people with health insurance without much considerations to benefit over risk of harm since this would mean more financial incentive.

Radiologists have a role to play to control MDCT overuse which include ensuring the efficient use of imaging resources, political advocacy with policy-makers to contain the rising cost of care and the role of a safety officer amongst others to reduce exposure to ionizing radiation [22]. However, this important role is not always possible to assume due to increasing workload and conflict of interest as often many more exams would mean an increase in the honorarium of the radiologist. Also, respect for the clinical judgment of a referring physician can also limit the radiologist´s role in determining the potential benefit of a given study over the risk of harm. Nevertheless, emphasis would have to be placed on justifying every single MDCT study irrespective of whether payments are made OOP or through a third party such as a health insurance scheme, to prevent the wanton exploitation of limited resources.

To further reduce the number of inappropriate MDCT scans, we suggest specialist physician authorization should be a requirement on request forms except in situations where imaging is urgent such as in trauma. For clients with health insurance, physicians working for such companies should be accountable for every MDCT request they authorize to be performed. Also, the use of guidelines such as the ACR Appropriateness Criteria® by referring physicians should be encouraged. With regards to training, medical trainees should be instructed on the risk of harm from ionizing radiation due to the use of some medical imaging studies and emphasis should be placed on identifying the most appropriate study to request for specific clinical conditions.

**Limitations:** reporting bias is a limitation to this study. In some situations all the fields of the data forms could not be filled for lack of accurate information. Also the finding of each individual MDCT scan was not correlated to appropriateness criteria to establish the diagnostic yield. Furthermore, a considerable percentage of MDCT scans could not be classified for appropriateness due to the absence of sufficient clinical information from the request forms and these technical information could not be provided by the participants. Referring healthcare providers could not be contacted for clinical information for their patients due to the absence of an address or telephone numbers on official stamps. It is therefore likely the excluded MDCT studies could have influenced the findings of the appropriateness analysis.

## Conclusion

MDCT use in the study setting cuts across all social strata with a considerable proportion of scans judged to be inappropriate. This indicates some lapses in the efficient use of resources and therefore the need for measures to optimize MDCT utilization so as to protect users from unnecessary expenditures and radiation exposure.

### What is known about this topic

- Multidetector computed tomography utilization is associated with high exposure to ionizing radiation;
- Multidetector computed tomography is used by people of all social strata.

### What this study adds

- The proportion of inappropriate multidetector computed tomography scans is considerable with increased risk of unnecessary exposure to ionizing radiation;
- Multidetector computed tomography over-utilization is an unintended consequence of health insurance ownership.
